# The Final Vindication

**Published:** 1924-12

**Authors:** 


THE
### HOSPITAL and HEALTH
REVIEW

### THE FINAL VINDICATION.

In writing last month upon the fifth annual report of the Joint Council of the Order of St. John and the British Red Cross Society on "The Voluntary Hospitals in Great Britain," we said that it formed "the vindication of Voluntaryism." The coping-stone has since been placed upon that vindication by the publication of the report upon the income and expenditure in 1923 of 116 London hospitals which has been prepared under the direction of the Hospital Economy Committee of King Edward's Hospital Fund for London. The most cursory examination of this extraordinarily interesting document makes it clear that, so far, at all events, as income is concerned, the voluntary hospitals have not only recovered from the heavy blows they received during and immediately after the War, but have happily started upon a career of renewed prosperity. This is not to say that they have all got everything that they need, and still less that the shadow of debt has for ever been chased away—hospitals in the mass are never likely to be in a position which would enable them to enjoy a comfortable, if inglorious, stagnation. Very great things have, however, been achieved. In 1920 the net aggregate deficit of the 116 institutions reported upon was £381,000; in 1921 this disquieting debit balance was reduced to £209,000; in 1922 there was a farther, if small improvement, and the deficit sank to £175,000. The year 1923 was a period of present comfort and future hope for the London hospitals. The legacies which fell in during the year amounted to close upon half-a-million, of which nearly a quarter of a million was received from a single source. The result was a surplus of £227,000 on the year, but even without this *aubaine* the deficit would have been brought down to £20,000. Nor is this the complete measure of the improvement. There has been not only a growth of income but a reduction of expenditure, which is considerably lower than in 1920. After having risen from £1,212,000 in 1913 to £2,813,000 in 1920, it began to fall, and in 1922 it was £2,590,000. Last year it rose again by £40,000, not because of any lack of economy. but because there was a growth in the volume of work done.

Encouraging as are the figures in the aggregate, and conclusively as they prove that the voluntary system has of late gained rather than lost in vitality, they must not be allowed to create the impression that the hospital position in London has become unassailable. While last year 62 of the 116 hospitals had surpluses amounting to £382,000, 54 had deficits totalling £115,000. Thus there were nearly as many with a balance on the wrong as on the right side. No can we expect that the progress of 1923 will continue at the same rate—huge legacies do not fall in regularly. There is every reason to hope and believe that public support will be on the ascending scale and, at the same time, that the efforts which are being made to reduce working costs will enable still more effective use to be made of the money.

Better and more economical administration and more scientific account-keeping were never so important to hospitals as to-day. They no longer draw their incomes from the comparatively restricted range of well-to-do benevolence. The regular weekly pence of the artisan have become as important to them as the annual or occasional cheque of the comfortable benefactor, and one of the most powerful appeals they can make to the community in the mass is the ability to show that their resources are carefully husbanded, and that their business management is not inferior to that of commercial concerns whose prosperity depends upon watchful care of finance and the stoppage of many small holes through which money may leak. The report of the Economy Committee contains many remarks and a mass of statistics relating to working costs which should be of the highest interest and the greatest practical moment to everyone concerned in hospital administration who realises the necessity of analysis and comparison in regard to every item of the expenditure of what are, in the most real sense, trust funds.

The necessity for larger income and improved methods of spending is vividly illustrated by the large increase in the volume of work done by the London hospitals since the year before the War. In 1923 there were 22,600 more in-patients than in 1913 and 37,000 more out-patients, while the increase in the attendances of out-patients in the same period was enormous—close upon a million and a half, indeed. It is highly probable that in the near future, as the services the hospitals can render to the country become more fully and widely recognised, these figures will swell to a substantial extent, with corresponding pressure upon their resources. There are, moreover, two serious circumstances to be borne in mind—the continued existence of accumulated deficits, and the growing need of building together with the ever-increasing demand for better equipment. Here, then, are abundant sources of concern for those who are responsible for the finance of hospitals. Happily the worst anxiety of all—the fear of the collapse of the voluntary principle and its replacement by some combined State and municipal system—no longer assails them. It has now been made clear that voluntaryism is equal to the demands upon it, and that the country infinitely prefers the old way of supporting institutions which every year make a larger demand upon its gratitude and admiration. English people have a rooted dislike of official intervention in directions where voluntary effort seems to them to be more seemly as well as more natural. Recent difficulties have done much more than deepen the charitable instinct. They have aroused at once a sense of duty and a determination that the sacred work of preventing and curing disease shall not become a mere affair of bureaucracy. Our hospitals lie very close to the heart of the English people, who, as we see, are readier than ever to tax themselves that they may pursue their beneficent work unhampered by any form of official interference.

